# Personality traits as behavioral determinants of road traffic injury risk: a mechanism-informed cross-sectional analysis

**DOI:** 10.3389/fpubh.2026.1842790

**Published:** 2026-06-03

**Authors:** Anderson Cheong, Nik Hisamuddin Nik Ab Rahman, Wan Hafiz Irshad Wan Mohd Zuhdi

**Affiliations:** 1 Surgical Department, Monash Health, Clayton, VIC, Australia; 2 Emergency Medicine, Universiti Sains Malaysia, Kubang Kerian, Malaysia

**Keywords:** agreeableness, big five personality, Malaysia, motor vehicle accident, neuroticism, personality trait, road traffic injury, trauma

## Abstract

**Background:**

Road traffic injuries (RTIs) remain a major global public health burden and a leading cause of mortality, particularly among individuals aged 5–29 years. While environmental and infrastructural determinants have been widely studied, the role of individual behavioral predispositions, including personality traits, remains underexplored. This study examines the association between the Big Five personality traits and RTI risk within a behavioral and injury prevention framework.

**Methods:**

A hospital-based cross-sectional study with a case–control analytical framework was conducted in two tertiary centers in Kelantan, Malaysia. Adults aged 18–60 years with RTIs were recruited from emergency departments, while controls were randomly selected from outpatient populations. Personality traits were assessed using the validated Bahasa Malaysia version of the Revised NEO Personality Inventory. A multivariable logistic regression analysis was performed to evaluate the associations between personality traits and RTI risk, adjusting for relevant confounders.

**Results:**

A total of 425 participants were included (229 RTI cases and 196 controls) in the study. The RTI group demonstrated marked male predominance. The distribution of personality traits was comparable between the groups. In adjusted analyses, agreeableness and neuroticism were significantly associated with an increased risk of RTIs [OR 1.12, 95% CI (1.03–1.21); OR 1.10, 95% CI (1.03–1.18), respectively].

**Conclusion:**

Personality traits may contribute to RTI risk through behavioral mechanisms involving emotional regulation, attention control, and decision-making. These findings support an integrated model of RTI risk incorporating both behavioral and environmental factors, with implications for targeted road safety and public health interventions.

## Introduction

Road traffic injuries (RTIs) account for approximately 1.2 million deaths and up to 50 million injuries each year worldwide, with projections suggesting a substantial increase over the next two decades (1–3). RTIs encompass collisions involving motor vehicles, non-motorized road users, pedestrians, and passengers (4, 5). Globally, RTIs remain the leading cause of death among individuals aged 5–29 years (6–8). In Malaysia, the World Health Organization estimates that approximately 5,000 deaths each year are caused by RTIs.

While extensive research has examined environmental, infrastructural, and behavioral determinants of RTIs, comparatively few studies have explored the role of individual personality traits in shaping injury risk (9–15). Existing literature remains heterogeneous, with inconsistent findings across populations and study designs. Notably, trait-specific behavioral pathways linking personality to RTI risk remain insufficiently characterized in real-world injury epidemiology. Addressing this gap is important for developing a more integrated understanding of RTIs that incorporates both external risk factors and individual behavioral predispositions.

From a theoretical perspective, personality traits can be situated within a neurocognitive framework, where individual differences in emotional processing, attention regulation, executive function, and reward sensitivity influence behavior in complex and dynamic environments such as road use. Neuroticism, for example, has been associated with heightened emotional reactivity and increased sensitivity to stress, potentially impairing decision-making under high-demand conditions. Individuals with high levels of neuroticism may be more susceptible to attention disruption, anxiety, and impulsive responses, thereby increasing their vulnerability to RTIs. In contrast, conscientiousness is associated with stronger executive control, effective planning, and adherence to rules, which may confer a protective effect through safer and more regulated driving behavior.

Within this framework, personality traits may influence RTI risk through distinct behavioral pathways. Neuroticism may increase this risk through stress-induced attention disruption and impaired cognitive control, whereas agreeableness—typically characterized by interpersonal compliance and a preference for social harmony—may contribute to RTI risk in situations requiring rapid, assertive decision-making. In dynamic traffic environments, these personality tendencies may manifest as delayed responses, increased susceptibility to distraction, or prioritization of social cues over task-focused attention.

This study examines the association between the Big Five personality traits—extraversion, neuroticism, agreeableness, openness, and conscientiousness—and RTI risk by comparing individuals with RTIs to a control population. We hypothesize that personality traits are significantly associated with RTI occurrence, with neuroticism and agreeableness representing key candidate traits linked to increased risk through mechanism-informed behavioral pathways. By situating these associations within a theoretical framework, this study seeks to contribute to a more comprehensive, behaviorally informed understanding of RTI risk within the Malaysian context. RTIs may therefore be conceptualized not only as environmental events but also as behaviorally mediated outcomes arising from the interaction between individual cognitive–emotional predispositions and dynamic traffic demands. Understanding the behavioral determinants of RTI risk has important implications for public health strategies, particularly for informing targeted interventions and risk stratification approaches for improving road safety.

## Methods

### Study design and setting

This study was a hospital-based cross-sectional analysis that used a case–control analytical framework. The participants in the test group were recruited from the emergency departments of two hospitals. The participants in the control group were randomly selected and recruited from the outpatient departments of the same hospitals. The study was conducted over the period of 10 months, between 31 January 2021 and 31 October 2021.

### Participant selection

For the case (RTI) group, participants were randomly selected from among patients who presented to the emergency departments during the study period, using a computer-generated randomization process until the required sample size was achieved. All individuals admitted to the emergency departments for road traffic injuries were screened as potential participants. Eligible individuals were aged 18–60 years. They provided informed consent, had no known underlying psychiatric illnesses, and sustained injuries classified with Abbreviated Injury Scale (AIS) scores ≤2. This threshold was applied to minimize the impact of severe injury, including potential cognitive impairment, on the reliability of the questionnaire. Consenting participants were monitored throughout their hospital stay and during follow-up visits to ensure ongoing eligibility and to document any clinical changes.

For the control group, participants were recruited from hospital outpatient departments using a similar randomization approach. Individuals aged 18–60 years who attended outpatient clinics were assigned random numbers and were selected using a computer-generated sequence. The eligibility criteria mirrored those for the case group, including the ability to provide informed consent and an absence of known psychiatric illnesses, to ensure comparability between the two groups. Illiterate individuals were excluded to maintain the validity of questionnaire-based assessments.

Outpatient attendees were selected as controls, as they represent a relatively stable, community-dwelling population without acute traumatic injury, allowing for the comparison of baseline personality traits in individuals not currently exposed to RTIs. This approach assumes that outpatient attendees come from the same source population as RTI cases, but are not currently exposed to the outcome of interest, thereby allowing for the valid estimation of exposure–outcome associations in a hospital-based case–control framework. Nevertheless, the use of outpatient controls may introduce selection bias, as healthcare-seeking individuals may differ systematically from the general population in terms of health behaviors or socioeconomic characteristics. This limitation was partially mitigated through random sampling and the application of consistent eligibility criteria across both groups; however, residual selection bias cannot be excluded.

Emergency physicians routinely classify trauma severity using the Abbreviated Injury Scale (AIS), which was used in this study to guide participant selection. Patients with AIS scores >2 were excluded due to the increased likelihood of significant injury, including traumatic brain involvement, which could impair comprehension and affect the reliability of questionnaire responses.

### Data collection

General demographic data, including age, ethnicity, and sex, were collected during the data collection phase using a structured, self-administered questionnaire. In addition, personality traits were assessed using the Revised NEO Personality Inventory (NEO-PI-R). A validated Bahasa Malaysia version of the instrument was utilized to minimize potential communication and interpretation bias, as previously described. The questionnaire was administered under the supervision of a trained investigator to ensure the completeness and consistency of responses.

### Inventory tool

Among the available personality assessment tools, the Big Five Personality model was selected due to its strong predictive validity, cross-cultural applicability, and relative stability throughout an individual’s lifespan. Personality traits were assessed using the NEO Personality Inventory (NEO-PI), which is a widely validated instrument for the measurement of the five major personality domains.

To ensure measurement accuracy and minimize potential communication or interpretation bias, an officially translated Bahasa Malaysia version of the Revised NEO Personality Inventory (NEO-PI-R) was utilized. This version, translated and validated by Haziq Azree Yazid, has undergone formal cultural adaptation and psychometric validation, demonstrating satisfactory reliability and construct validity within the Malaysian population. The use of a locally validated instrument enhances the cultural relevance and interpretability of the findings while maintaining consistency with the original framework established by Paul Costa and Robert McCrae (1995).

### Bias minimization

Selection bias was mitigated using a computer-generated randomization process for participant recruitment. Random error was minimized by using a sufficiently large sample size. Assessment bias was further reduced by employing trained clinicians to evaluate injury severity using standardized scoring systems.

### Sample size

The sample size was calculated using an online tool for estimating a single proportion (Statulator: statulator.com/SampleSize/ss1P.html). A confidence level of 95% and a margin of error of 5% were applied. The minimum required sample sizes were estimated at 191 participants for the case group and 96 participants for the control group. To account for an anticipated non-response rate of 20%, the total required sample size was increased to 344 participants.

### Statistical analysis

The data were initially tabulated in Microsoft Excel 2021 and subsequently processed and analyzed using the Statistical Package for the Social Sciences (SPSS), version 28. Age was treated as a continuous variable. Socioeconomic status was categorized as low, middle, or high based on the Household Income Survey Reports from the Department of Statistics Malaysia. Sex and ethnicity were analyzed as categorical variables.

An independent t-test was performed to assess differences in mean values between RTIs and the control groups, assuming no association between personality traits and RTI status. Model adequacy was evaluated using the omnibus test before further analysis. The inclusion of the five personality trait components improved the overall model fit and demonstrated acceptable reliability.

A multivariable logistic regression analysis was conducted to examine the association between personality traits (independent variables) and RTI status (dependent variable), while adjusting for potential confounders. Covariates included age, sex, and socioeconomic status. Odds ratios with corresponding 95% confidence intervals were calculated to quantify the strength and direction of these associations. No missing data were identified during the data cleaning process.

## Ethical approval

This study was approved by the Ethics Committee of Universiti Sains Malaysia (Jawatankuasa Etika Penyelidikan Manusia, USM/JEPeM/20110563). This study was conducted in accordance with the Declaration of Helsinki. There were no competing interests or funding. All participants received detailed information regarding the study’s objectives and procedures before providing their written informed consent. All participants provided consent to the publication of the study. The dataset can be obtained from the corresponding author upon reasonable request.

## Results

A total of 425 participants were recruited from two hospitals, comprising 229 individuals with RTIs and 196 controls. These cohorts constituted the RTI group and the control group, respectively.

Within the RTI group, the majority of participants (85%) were of Malay ethnicity, consistent with the demographic distribution of Kelantan. The majority of participants were aged between 20 and 30 years, with fewer individuals in older age groups. The mean age of the RTI group was 38.5 years (SD 13.9), compared to a younger mean age in the control group (31.9 years, SD 12.4). There was a marked male predominance, with a male-to-female ratio of approximately 4:1 in the RTI group.

Initial comparisons between the RTI and control groups using independent t-tests did not demonstrate statistically significant differences in the general study model (Table 1). Subsequently, a multivariable logistic regression model was used to examine the association between personality traits and RTI risk, adjusting for potential confounding variables.

**Table 1:** Comparison of demographic characteristics and Big Five personality trait scores between participants with and without road traffic injury (RTI).

	Non – RTI (*n* = 196)	RTI (*n* = 229)	Mean difference	*t*-test	Odds Ratio	
	Mean ± SD	Mean ± SD		*p*-value		*p*-value
Sex	-	-	-		0.34	0.00
Age	31.9±12.4	38.5 ± 13.9	6.6	0.00	0.91	0.54
Conscientiousness	30.5 ± 3.5	30.1 ± 4.4	0.4	0.36	1.03	0.41
Extraversion	23.3 ± 3.5	23.8 ± 3.3	0.5	0.29	1.05	0.19
Agreeableness	31.2 ± 3.1	32.2 ± 3.9	1.0	0.67	1.12	0.01
Neuroticism	18.8 ± 3.9	20.8 ± 4.8	2.0	0.20	1.10	0.00
Openness	30.9 ± 3.8	31.9 ± 4.3	1.0	0.97	1.02	0.11

Descriptive analyses demonstrated that personality trait distributions were similar across groups, suggesting that the observed differences in RTI risk are unlikely to be driven by gross trait imbalance but rather by differential effects of specific traits. Agreeableness was the most prominent trait in both groups, followed by openness, conscientiousness, extraversion, and neuroticism. Model fit was assessed using the likelihood ratio χ^2^ test, which supported the adequacy of the model. The Wald χ^2^ test indicated that age, sex, agreeableness, and neuroticism were significantly associated with RTI status.

In the multivariable logistic regression analysis, agreeableness and neuroticism remained statistically significant predictors of RTI risk. A one-point increase in the agreeableness score was associated with higher odds of RTIs [OR 1.12, 95% CI (1.03–1.21)], while neuroticism was associated with increased odds of RTIs [OR 1.10, 95% CI (1.03–1.18)]. Effect estimates were reported with 95% confidence intervals to reflect statistical precision; wider intervals observed in smaller subgroups indicate reduced precision of the estimates. These results identify neuroticism and agreeableness as trait-level markers of behavioral vulnerability rather than as isolated statistical predictors.

These findings identify agreeableness and neuroticism as candidate personality traits associated with RTI risk, warranting further investigation within a mechanism-informed behavioral risk framework.

## Discussion

The study population demonstrated a marked male predominance, consistent with global epidemiological patterns of road traffic injuries (RTIs), particularly in low- and middle-income settings. This likely reflects greater exposure to high-risk road use among men, including occupational driving and motorcycle use. While the statistical analyses were adjusted for sex, the findings may be more applicable to male road users, and caution is warranted when extrapolating the results to female populations. Future studies with more balanced sex distributions or sex-stratified analyses are needed to better delineate sex-specific risk profiles and injury patterns.

The present findings provide evidence of a mechanism-informed relationship between personality traits and RTI risk, suggesting that individual behavioral predispositions contribute meaningfully to injury occurrence. Each one-point increase in neuroticism score was associated with an approximately 10% increase in the likelihood of RTIs (*p* < 0.05), while agreeableness was associated with a 12% increase per unit score (p < 0.05). Although these per-unit effect sizes appear modest, their impact may be amplified at the population level due to the continuous distribution of personality traits. These findings suggest that even modest trait-level differences may translate into meaningful population-level risk through cumulative behavioral effects over repeated traffic exposure. Nevertheless, the cross-sectional comparative design precludes causal inference, and prospective cohort studies are required to further elucidate temporal and causal relationships.

Importantly, these findings extend the existing RTI literature by shifting the focus from predominantly environmental and infrastructural determinants toward an integrated model that incorporates individual behavioral predispositions. By linking personality traits to plausible cognitive–behavioral mechanisms, this study suggests that injury risk may be partly mediated by stable behavioral tendencies that influence decision-making in dynamic traffic environments. This perspective aligns with contemporary injury epidemiology frameworks that emphasize the interaction between human factors and situational demands and highlights the relevance of incorporating behavioral risk profiling into road safety and public health strategies.

From a theoretical perspective, these associations can be understood within a mechanism-based behavioral framework. Neuroticism is characterized by heightened emotional reactivity, sensitivity to stress, and susceptibility to negative emotions (16, 17). In the context of driving—a task that requires sustained attention and rapid decision-making—these traits may predispose individuals to stress-induced attention disruption and impaired executive control (18). Individuals with high levels of neuroticism are more likely to experience anxiety, frustration, and mind-wandering in challenging traffic conditions, which may divert cognitive resources away from situational awareness and negatively impact decision-making (19). These mechanisms are consistent with models of cognitive overload and affect-driven impairment in high-demand environments, providing a plausible pathway link between neuroticism and an increased risk of RTIs (20, 21).

The observed association between agreeableness and increased RTI risk is less intuitive and should be interpreted cautiously. While agreeableness is generally characterized by prosocial tendencies such as empathy, cooperation, and conflict avoidance, its role in dynamic traffic environments may be more complex. One plausible interpretation is that highly agreeable individuals may exhibit increased interpersonal compliance or reduced assertiveness, which could lead to delayed or suboptimal decision-making in situations requiring rapid, decisive responses (22, 23). Additionally, emerging evidence suggests that higher agreeableness may be associated with behaviors, such as using mobile phones while driving, potentially reflecting heightened responsiveness to social cues at the expense of task-focused attention (24). These findings suggest that the effect of agreeableness on driving behavior may not be uniformly protective; rather, it varies according to the demands of the situation, particularly in high-complexity or ambiguous traffic environments. Research on behavioral decision-making suggests that agreeableness may be associated with context-dependent reductions in self-regulatory control under time pressure, potentially shifting decision-making toward more automatic or affect-driven responses in complex driving environments (25). However, these behavioral mechanisms were not directly measured in the present study and, therefore, should be considered hypothesis-generating, identifying agreeableness as a potential behavioral determinant of RTI risk and proposing plausible mechanistic pathways that warrant further empirical investigation. Future studies that incorporate direct behavioral measures and prospective designs are required to test these hypotheses and clarify the underlying mechanisms.

Collectively, these findings suggest that personality traits may influence RTI risk by affecting emotional regulation, attention control, and social decision-making in traffic contexts. Variation in trait expression may affect reaction time, risk perception, and cognitive resilience under stress, thereby shaping behavioral responses during road use. Framing RTIs within this mechanism-informed perspective shifts the focus from purely environmental determinants toward an integrated model that incorporates individual behavioral predispositions.

However, these findings should be interpreted with caution. The cross-sectional design limits causal inference, and the results are inherently context-specific. While statistically significant associations were identified, they should be regarded as preliminary and hypothesis-generating rather than definitive. Future research using prospective, multi-center designs and incorporating direct behavioral measures is necessary to validate these associations, establish causal pathways, and enhance generalizability.

### Limitations

This study has several limitations that warrant consideration when interpreting the findings. First, it was conducted within a single state (Kelantan, Malaysia), with a study population predominantly of Malay ethnicity. As such, the external validity of the findings may be limited, and caution is required when generalizing to populations with different demographic, cultural, or road-use characteristics.

Second, the cross-sectional design constrains causal inference because the temporal relationship between personality traits and RTI risk cannot be established. Accordingly, the observed associations should be interpreted as indicative of potential behavioral relationships rather than definitive causal effects.

In addition, the relative paucity of comparable studies limits direct contextualization within the existing literature. However, this also underscores the exploratory and hypothesis-generating contribution of the present study in an under-researched area. The sample size was adequate to support the statistical robustness of the observed associations, enhancing the internal validity of the findings despite these contextual limitations.

Finally, the applied inclusion and exclusion criteria resulted in the exclusion of certain patient groups, including individuals with severe injuries, low Glasgow Coma Scale scores, and limited literacy. This may restrict the applicability of the findings across the full clinical spectrum of RTI presentations, particularly in more severe cases where cognitive and behavioral responses may differ.

Despite these limitations, the study provides a novel, mechanism-informed perspective on RTI risk using a validated personality framework within a real-world clinical population. Collectively, these limitations restrict causal inference, reduce external generalizability, and constrain definitive interpretation of the proposed behavioral mechanisms.

## Conclusion

This study demonstrates that personality traits, particularly neuroticism and agreeableness, are associated with RTI risk through plausible behavioral mechanisms involving emotional regulation, attention control, and social decision-making. These findings support an integrated model of RTI risk in which individual behavioral predispositions interact with environmental demands. Incorporating psychological factors into road safety frameworks may enhance prevention strategies. Future research should prioritize prospective designs and direct behavioral measurement to validate these pathways and inform targeted, evidence-based public health interventions.

## Data Availability

The original contributions presented in the study are included in the article/supplementary material; further inquiries can be directed to the corresponding author.
